# Hrd1-dependent Degradation of the Unassembled PIGK Subunit of the GPI Transamidase Complex

**DOI:** 10.1247/csf.21019

**Published:** 2021-06-30

**Authors:** Kohei Kawaguchi, Miki Yamamoto-Hino, Yoshiko Murakami, Taroh Kinoshita, Satoshi Goto

**Affiliations:** 1 Department of Life Science, Rikkyo University, 3-34-1 Nishi-Ikebukuro, Toshima-ku, Tokyo 171-8501, Japan; 2 Research Institute for Microbial Diseases, Osaka University, Suita, Osaka 565-0871, Japan

**Keywords:** Glycosylphosphatidylinositol, GPI transamidase complex, protein stability, transamidation, ERAD

## Abstract

Glycosylphosphatidylinositol (GPI)-anchored proteins are post-transcriptionally modified with GPI and anchored to the plasma membrane. GPI is attached to nascent proteins in the endoplasmic reticulum by the GPI transamidase complex, which consists of PIGT, PIGK, GPAA1, PIGU, and PIGS. Of these, PIGK is a catalytic subunit that is unstable without PIGT. This study investigated the pathway by which unassembled PIGK not incorporated into the complex is degraded. We showed that unassembled PIGK was degraded via the proteasome-dependent pathway and that Hrd1 (also known as SYVN1), a ubiquitin ligase involved in the endoplasmic reticulum-associated degradation pathway, was responsible for degradation of unassembled PIGK.

## Introduction

Glycosylphosphatidylinositol (GPI)-anchored proteins (GPI-APs) are post-translationally modified with GPI and anchored to the plasma membrane. GPI-anchoring regulates various cellular molecules including hydrolytic enzymes, adhesion molecules, receptors, protease inhibitors, and complement regulatory proteins. Therefore, defective GPI-APs can cause serious diseases, including inherited GPI deficiency and paroxysmal nocturnal hemoglobinuria (Kinoshita, 2020).

GPI is attached to nascent proteins in the endoplasmic reticulum (ER) by the GPI transamidase complex (GPI-TA). The GPI-TA consists of at least five subunits called PIGT, PIGK, GPAA1, PIGU, and PIGS. PIGK has a catalytic activity. It cleaves the C-terminal GPI signal of the precursor protein and forms an enzyme-substrate complex via a thioester bond (Ohishi *et al.*, 2000). GPAA1 is predicted to form an amide linkage between the newly exposed C-terminal amino acid of the precursor protein and the phosphoethanolamine of GPI (Eisenhaber *et al.*, 2014). GPAA1 and PIGU are suggested to be subunits that recognize GPI (Eisenhaber *et al.*, 2018; Hong *et al.*, 2003; Vainauskas and Menon, 2004). Although PIGS is essential for the GPI modification, its specific role is unknown. PIGT is required for complex assembly and protein stability of GPAA1 and PIGK in mammals (Ohishi *et al.*, 2001). A disulfide bond forms between PIGT and PIGK in mammals (Ohishi *et al.*, 2003) .

Many proteins assemble into complexes. The stoichiometry of the subunits is tightly regulated to suppress proteotoxic effects. (Juszkiewicz and Hegde, 2018) It is unknown how the stoichiometry of the GPI-TA subunits is regulated. We previously revealed that PIGK is destabilized in the absence of any other GPI-TA subunit in *Drosophila* (Kawaguchi *et al.*, 2019). Similarly, efficient expression of exogenous PIGK requires co-transfection of PIGT in mammals (Ohishi *et al.*, 2001). These findings suggest that the level of PIGK is tightly regulated and that unassembled PIGK not incorporated into the GPI-TA is eliminated. This study investigated the pathway by which unassembled PIGK is degraded in mammalian cells. We revealed that unassembled PIGK is degraded via the Hrd1-dependent ER-associated degradation (ERAD) pathway.

## Materials and Methods

### Cell culture and DNA transfection

HEK293 cells and variants were grown in Dulbecco’s modified Eagle’s medium supplemented with 10% fetal bovine serum. DNA was transfected using polyethylenimine transfection reagents (Polyscience, Warrington, PA) following the manufacturer’s instructions.

### Plasmids

For CRISPR-based gene knockout (KO), BbsI-digested pX330 was ligated with a DNA fragment designed using the CRISPOR website (http://crispor.tefor.net/). The following oligonucleotides were used for gene KO: PIGK-gRNA1-F1, caccGCTCTTGTCCTTCGGCAGCG; PIGK-gRNA1-R1, aaacCGCTGCCGAAGGACAAGAGc; Doa10-gRNA1-F1, caccgTGTAGAGTGTGTCGGTCAGA; Doa10-gRNA1-R1, aaacTCTGACCGACACACTCTACAc; Doa10-gRNA2-F1, caccgATACTGCCAGTACATACACA; Doa10-gRNA2-R1, aaacTGTGTATGTACTGGCAGTATc; Hrd1-gRNA1-F1, caccGGCCAGGGCAATGTTCCGCA; Hrd1-gRNA1-R1, aaacTGCGGAACATTGCCCTGGCc; Hrd1-gRNA2-F1, caccgTGGTCAGGTACACCACAGTG; Hrd1-gRNA2-R1, aaacCACTGTGGTGTACCTGACCAc; gp78-gRNA1-F1, caccgCGTTAGCTGGTCCGGCTCGC; gp78-gRNA1-R1, aaacGCGAGCCGGACCAGCTAACGc; gp78-gRNA2-F1, caccgGCCTCGCGAACGGCGCCCAC; gp78-gRNA2-R1, aaacGTGGGCGCCGTTCGCGAGGCc; TRC8-gRNA1-F1, caccgGCACGATGCAGAACCGGCTT; TRC8-gRNA1-R1, aaacAAGCCGGTTCTGCATCGTGCc; TRC8-gRNA2-F1, caccgGAACAGGGTACCGTACGTCC; TRC8-gRNA2-R1, aaacGGACGTACGGTACCCTGTTCc; RHBDL4-gRNA1-F1, caccgCCCTCTTGATCTCCGTTGCA; RHBDL4-gRNA1-R1, aaacTGCAACGGAGATCAAGAGGGc; RHBDL4-gRNA2-F1, caccgAGCAATGGGAAAACGTACTC; and RHBDL4-gRNA2-R1, aaacGAGTACGTTTTCCCATTGCTc.

Wild-type and C329S mutant Hrd1 and PIGK cDNA fragments were integrated into the pIRES2-3xFLAG-ZsGreen1 and pIRES2-3xMyc-ZsGreen1 vectors, respectively, using an InFusion HD cloning kit (Takara Bio, Shiga, Japan). Hrd1 cDNA fragments were amplified from pcDNA6-Hrd1-WT/C329S-myc-His, which were kindly provided by Masayuki Kaneko (Hiroshima University, Japan). PIGK cDNA fragments were amplified from cDNA derived from SH-SY5Y cells.

### Generation of KO cell lines

To generate gene KO cell lines, HEK293 and HEK293-CD16-PIGT-KO cells were transiently transfected with one or two pX330 plasmids bearing the sgRNA sequence targeting each gene, and clonal KO cells were obtained. KO of PIGK was validated by flow cytometry to confirm the loss of GPI-AP expression using the fluorescent-labeled inactive toxin aerolysin (FLAER)-Alexa 488 probe. KO of Hrd1, gp78, TRC8, and Doa10 was validated by PCR to confirm a large deletion of each gene. KO of gp78 was validated by immunoblotting to confirm the loss of gp78 protein.

### Immunoblotting

Cells were lysed in lysis buffer (20 mM Tris-HCl, pH 7.4, 150 mM NaCl, 1% Nonidet P-40, and 1 mM EDTA) supplemented with a protease inhibitor cocktail (Nacalai Tesque, Kyoto, Japan), and subjected to SDS-PAGE following standard procedures. Proteins were transferred to PVDF membranes (Merck Millipore, Burlington, MA), which were then blocked with phosphate-buffered saline (PBS) containing 2% skimmed milk and incubated overnight with a primary antibody. The primary antibodies used were an anti-PIGK antibody (1:2000; rabbit; ab201693; Abcam, Cambridge, MA), an anti-myc antibody (1:2000; rabbit; A-14; Santa Cruz Biotechnology, Dallas, TX), an anti-FLAG antibody (1:2000; mouse; 1E6; Wako Biochemicals, Osaka, Japan), an anti-gp78 antibody (1:2000; rabbit; #9590; Cell Signaling Technology, Beverly, MA), and an anti-GAPDH antibody (1:10000; rabbit; NB100-56875; Novus Biologicals, Littleton, CO). After three washes for 5 min with PBS containing 0.05% Tween 20, the primary antibody was detected with an HRP-conjugated anti-mouse IgG or anti-rabbit IgG antibody (1:10000; Jackson ImmunoResearch, West Grove, PA). Signals were visualized using Clarity Western ECL Substrate (Bio-Rad, Irvine, CA) and the ImageQuant LAS 4000 mini chemiluminescence detection system (GE Healthcare, Menlo Park, CA).

### Flow cytometry

HEK293 cells stably expressing PIGK-myc were harvested with trypsin/EDTA and fixed with phosphate buffer containing 2% paraformaldehyde. Fixed cells were permeabilized with PBS containing 0.05% Triton X-100 and stained with an anti-myc antibody (1:2000; mouse; PL14; MBL, Nagoya, Japan) for 15 min on ice. HEK293 cells transiently expressing PIGK-myc were harvested with trypsin/EDTA and stained with an anti-CD59 antibody (1:200; mouse; H19; BD Biosciences, San Jose, CA). After incubation, cells were washed twice with FACS buffer (PBS containing 1% bovine serum albumin and 0.1% NaN_3_) and stained with APC-conjugated anti-mouse IgG for 15 min on ice. Finally, cells were washed twice with FACS buffer and analyzed with a FACSVerse flow cytometer (BD Biosciences). The resulting data were analyzed with FlowJo software (BD Biosciences).

### Reverse-transcription quantitative PCR (RT-qPCR)

Total RNA was extracted from cells using an RNeasy Kit (Qiagen, Hilden, Germany) according to the manufacturer’s protocol. Superscript III reverse transcriptase (Invitrogen; Thermo Fisher Scientific, Waltham, MA) and oligo(dT) primers were used for reverse transcription. Real-time PCR was performed using QuantStudio 12K Flex (Applied Biosystems; Thermo Fisher Scientific, Waltham, MA) and Power SYBR Green (Applied Biosystems). The amount of amplified transcript was calculated using the relative standard curve method and normalized against GAPDH as an internal control. The following primers were used: PIGK-qPCR-F1, CGTGGCCGCTAGTCATATCG; PIGK-qPCR-R1, CGGGATGTACACACCAGAACA; hGAPDH-qPCR-F1, TGCACCACCAACTGCTTAGC; and hGAPDH-qPCR-R1, GGCATGGACTGTGGTCATGAG.

## Results

### PIGT is required for PIGK expression

**Fig. 1:**
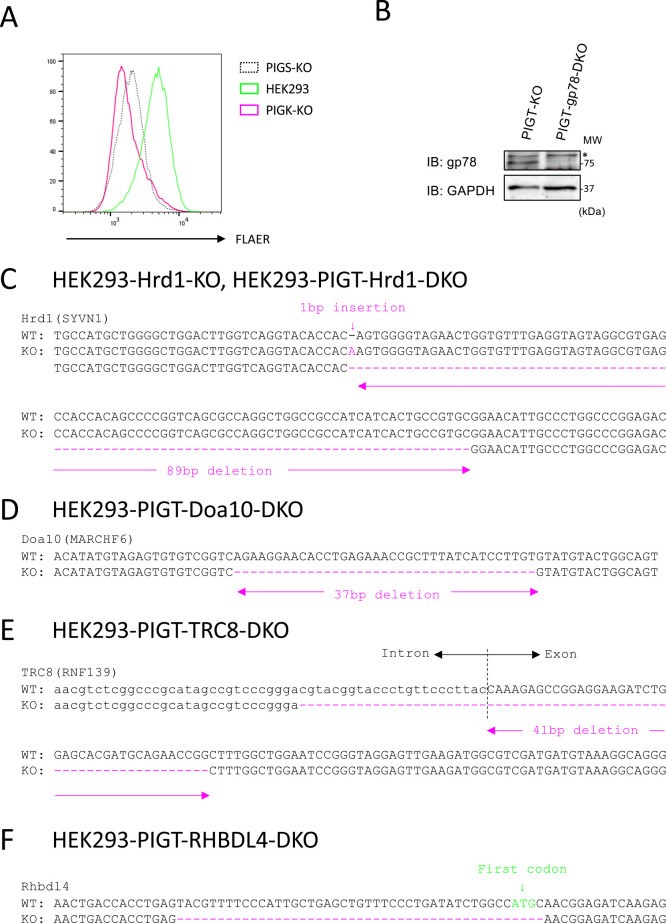
Knock out validation. (A) HEK293 (green line), HEK293-PIGS-KO (black dotted line), and HEK293-PIGK-KO (magenta line) cells were stained with FLAER-Alexa 488. (B) Lysates of HEK293-PIGT-KO and HEK293-PIGT-gp78-DKO cells were subjected to immunoblot (IB) analysis using the indicated antibodies. MW, molecular weight. The asterisk indicates a non-specific band. (C–F) The genome regions flanking the gRNA-targeted sites were amplified by PCR. A frameshift mutation was validated by Sanger sequencing. The same mutations were introduced in PIGT-Doa10-DKO, PIGT-TRC8-DKO, and PIGT-RHBDL4-DKO cells.

**Fig. 2:**
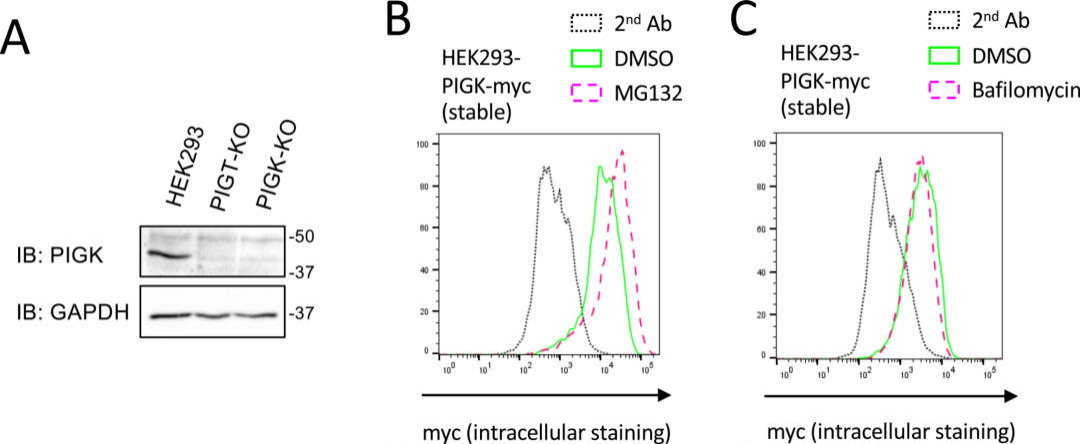
Identification of the pathway by which excess PIGK is degraded. (A) Lysates of HEK293, HEK293-PIGT-KO, and HEK293-PIGK-KO cells were subjected to immunoblot (IB) analysis using anti-PIGK and anti-GAPDH antibodies. (B, C) HEK293 cells stably expressing PIGK-myc were treated with 5 μM MG132 for 8 h (B) or 100 nM Bafilomycin A1 for 16h (C). The cells were fixed and permeabilized. Fixed cells were stained with an anti-myc antibody and subjected to flow cytometry. The black dotted line indicates cells stained only with the secondary antibody. The green line indicates cells treated with DMSO for 8 h (B) or 16 h (C). The magenta dashed line indicates cells treated with MG132 (A) or Bafilomycin A1 (B).

A previous study showed that expression of exogenous PIGK is inefficient and is enhanced by co-transfection of PIGT (Ohishi *et al.*, 2001). We sought to confirm that expression of endogenous PIGK requires PIGT. We knocked out PIGK in HEK293 cells. To validate the establishment of PIGK-KO cells, we stained these cells with FLAER, which binds specifically to GPI-APs. The level of GPI-APs on the surface of PIGK-KO cells was decreased and was similar to that on the surface of PIGS-KO cells (Fig. 1A). Lysates of parental, PIGT-KO, and PIGK-KO cells were subjected to immunoblotting with an anti-PIGK antibody (Fig. 2A). The band that migrated at approximately 45 kDa in parental cells was absent in PIGK-KO cells, suggesting that it represents endogenous PIGK. KO of PIGT reduced expression of PIGK to a similar level as that in PIGK-KO cells. This result suggests that expression of endogenous PIGK requires PIGT.

### Unassembled PIGK is degraded in a proteasome-dependent manner

Next, we investigated the pathway by which unassembled PIGK is degraded. We first established HEK293 cells stably expressing PIGK tagged with myc (HEK293-PIGK-myc). We hypothesized that excess PIGK is degraded via the proteasome- or lysosome-dependent pathway. To investigate this, we treated HEK293-PIGK-myc cells with MG132 and Bafilomycin, which inhibit proteasome activity and lysosome acidification, respectively. The cells were permeabilized, stained with an anti-myc antibody, and subjected to flow cytometry. Treatment with MG132, but not with Bafilomycin, increased the level of PIGK-myc (Fig. 2B, C). These results indicate that unassembled PIGK is degraded in a proteasome-dependent manner.

### PIGK is degraded in a Hrd1-dependent manner in PIGT-KO cells

**Fig. 3:**
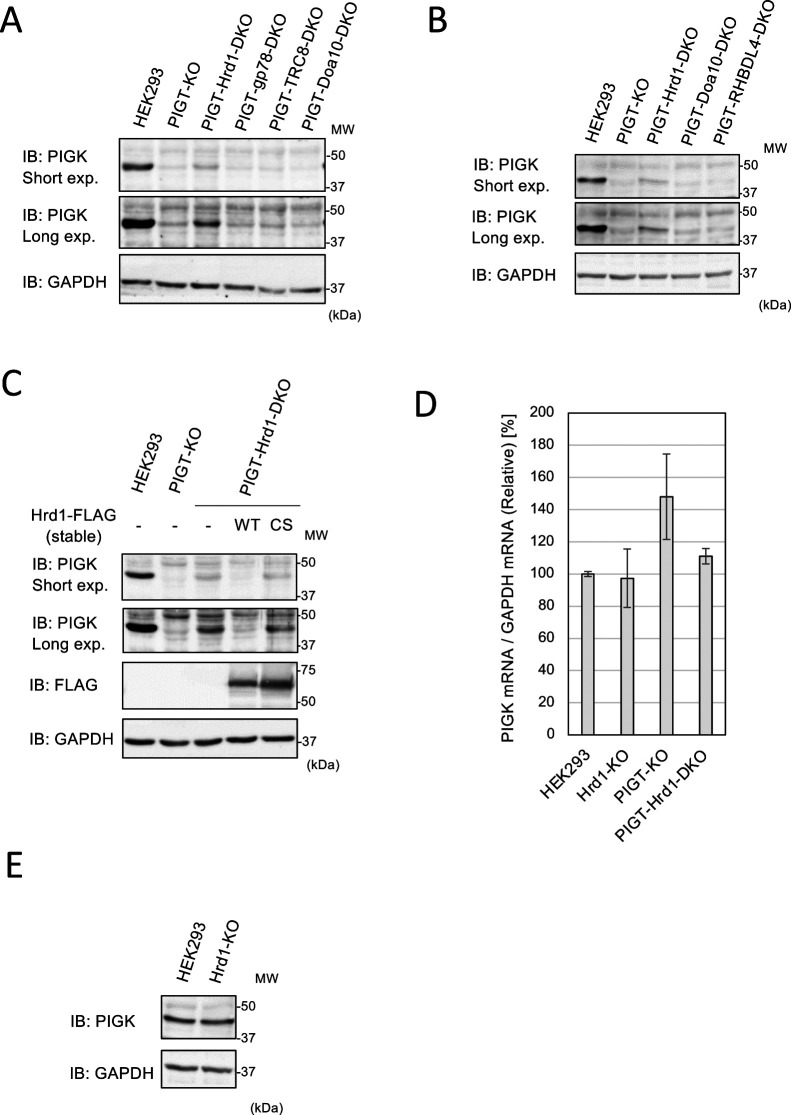
Identification of the ubiquitin ligase responsible for degradation of unassembled PIGK. (A, B, C, E) Lysates of HEK293 and the indicated derivative cells were subjected to immunoblot (IB) analysis using the indicated antibodies. Saturated pixels in long exposure images of IB: PIGK are shown in Fig. S1. WT, wild-type; CS, C329S; MW, molecular weight. (D) Total RNA extracted from parental, Hrd1-KO, PIGT-KO, and PIGT-Hrd1-DKO HEK293 cells was subjected to RT-qPCR. The relative mRNA levels of PIGK compared with that in parental cells are shown as bars. Error bars show the standard deviation. n=3.

We next investigated which E3 ubiquitin ligase is responsible for degradation of PIGK dissociated from PIGT. The GPI-TA mediates GPI anchoring in the ER; therefore, we hypothesized that unassembled PIGK is degraded via the ERAD machinery. We focused on four well-characterized ubiquitin ligases that are part of the ERAD machinery, namely, Hrd1 (also known as SYVN1), gp78 (also known as AMFR), TRC8 (also known as RNF139), and Doa10 (also known as MARCHF6) (Leto *et al.*, 2019; Stefanovic-Barrett *et al.*, 2018). We knocked out these four genes in PIGT-KO cells (Fig. 1B–E). These cells were subjected to immunoblotting with an anti-PIGK antibody. KO of Hrd1, but not of gp78, TRC8, or Doa10, restored expression of PIGK in PIGT-KO cells (Fig. 3A).

A recent study revealed that the intramembrane protease RHBDL4 is required for ERAD-dependent degradation of the unassembled STT3A subunit of the oligosaccharyltransferase complex (Knopf *et al.*, 2020). Therefore, we knocked out RHBDL4 in PIGT-KO cells (Fig. 1F). KO of RHBDL4 did not restore expression of PIGK in PIGT-KO cells (Fig. 3B).

To exclude the possibility that an off-target effect is responsible for the restoration of PIGK expression, we established PIGT-Hrd1-double knockout (DKO) cells stably expressing wild-type (WT) and inactive mutant (C329S) Hrd1. Expression of WT Hrd1, but not of C329S Hrd1, reduced expression of PIGK in PIGT-Hrd1-DKO cells (Fig. 3C). These results confirm that the ubiquitin-ligase activity of Hrd1 is required to reduce expression of PIGK in PIGT-KO cells.

To investigate whether KO of Hrd1 affects mRNA expression of PIGK, we quantified PIGK mRNA expression in parental, Hrd1-KO, PIGT-KO, and PIGT-Hrd1-DKO cells (Fig. 3D). mRNA expression of PIGK was comparable in parental, Hrd1-KO, and PIGT-Hrd1-DKO cells. PIGT-KO cells expressed a slightly higher level of PIGK mRNA. These results suggest that KO of Hrd1 restored expression of PIGK in PIGT-KO cells at the protein level.

To investigate whether Hrd1 regulates the expression level of PIGK in cells expressing PIGT, lysates of parental and Hrd1-KO cells were subjected to immunoblotting with an anti-PIGK antibody (Fig. 3E). Parental and Hrd1-KO cells expressed a similar level of PIGK. This result indicates that Hrd1 does not regulate the protein level of PIGK in cells expressing PIGT.

## Discussion

This study revealed that excess PIGK was degraded in a proteasome-dependent manner (Fig. 2B). In PIGT-KO cells, endogenous PIGK was degraded in a Hrd1-dependent manner (Fig. 3A, C). KO of Hrd1 did not increase the mRNA level of PIGK (Fig. 3D). Moreover, KO of Hrd1 did not increase the level of PIGK in cells expressing PIGT (Fig. 3E). Taken together, we conclude that the Hrd1-dependent ERAD pathway is required for degradation of unassembled PIGK, but not of PIGK incorporated into the GPI-TA.

Hrd1 forms a complex by interacting with accessory factors. Specifically, Hrd1 interacts with OS-9 and XTP3-B via SEL1L. OS-9 and XTP3-B recognize the mannose-trimmed N-glycan of misfolded proteins and recruit these proteins to the Hrd1-SEL1L complex for degradation (Hosokawa *et al.*, 2009, 2008). PIGK does not harbor the N-X-S/T consensus sequence that is required for N-glycosylation. Therefore, Hrd1 may recognize unassembled PIGK in an OS-9/XTP3-B-independent manner.

T cell receptor alpha (Tα) is a well-characterized ERAD substrate. Tα harbors a single transmembrane domain containing a charged residue that functions as a degradation signal. Normally, the transmembrane domain is masked by a binding partner. However, the transmembrane domain of unassembled Tα is exposed and functions as a degradation signal for the ERAD pathway (Bonifacino *et al.*, 1990). Therefore, the exposed transmembrane domain of PIGK may function as a degradation signal for the Hrd1-dependent ERAD pathway.

KO of Hrd1 did not completely restore the protein level of PIGK in PIGT-KO cells (Fig. 3A). Therefore, we cannot exclude the possibility that a factor other than Hrd1 is also involved in degradation of unassembled PIGK. A recent study revealed that the Asi1/Asi2/Asi3 E3 ligase complex is required for degradation of unassembled Gpi8 (ortholog of PIGK) and Gpi16 (ortholog of PIGT), whereas the contribution of Hrd1 and Doa10 is minor in yeast(Natarajan *et al.*, 2020). Mammalian homologs of Asi1, Asi2, and Asi3 have not been identified. The functional homologs of these proteins may be involved in degradation of unassembled PIGK.

A recent study showed that the intramembrane protease RHBDL4 cleaves the unassembled STT3A subunit of the oligosaccharyltransferase complex. This cleavage is required for ERAD-dependent degradation of unassembled STT3A (Knopf *et al.*, 2020). We showed that RHBDL4 was not required for degradation of unassembled PIGK (Fig. 3B). A further study is required to investigate the involvement of a factor other than Hrd1 in degradation of unassembled PIGK.

The amount of PIG-K was not increased in Hrd1-KO cells (Fig. 3E), indicating that PIG-K is not unassembled in normally growing cells and that the Hrd1-ERAD system does not contribute to the elimination of unassembled PIG-K from cells in such situations. Aneuploidy is a hallmark of cancer cells (Kolodner *et al.*, Science, 2011). An increased number of PIGK loci may require the elimination of unassembled PIGK. The Hrd1-ERAD system may degrade unassembled PIGK to relieve ER stress in cancer cells. If ERAD does not degrade unassembled PIG-K in such a situation, the exposed transmembrane domain may harm other proteins, resulting in cell death because of ER stress.

Unbalanced stoichiometry of the GPI-TA is believed to be involved in the onset and progression of cancer. PIGT, PIGU, GPAA1, and PIGS are overexpressed in many cancers, whereas PIGK is downregulated (Nagpal *et al.*, 2008). It is unknown how the stoichiometry of the GPI-TA is regulated. This study showed that the ERAD machinery is involved in regulation of this stoichiometry. The ERAD machinery may prevent the onset and progression of cancer by regulating the stoichiometry of the GPI-TA.

## Conflict of interest

The authors declare that there are no conflicts of interest.

## Abbreviations

DKO: double knockout
ER: endoplasmic reticulum
ERAD: endoplasmic reticulum-associated degradation
FLAER: fluorescent-labeled inactive toxin aerolysin
GPI: glycosylphosphatidylinositol
GPI-AP: glycosylphosphatidylinositol-anchored protein
GPI-TA: glycosylphosphatidylinositol transamidase complex
KO: knockout
PBS: phosphate-buffered saline
RT-qPCR: reverse-transcription quantitative PCR
Tα: T cell receptor alpha
WT: wild-type
