# Supplementary figures and images for: Hrd1-dependent Degradation of the Unassembled PIGK Subunit of the GPI Transamidase Complex
Source: Cell Struct Funct. 2021 Jun 30;46(2):65–71. doi: 10.1247/csf.21019 (PMC10511060; doi:10.1247/csf.21019)

**A**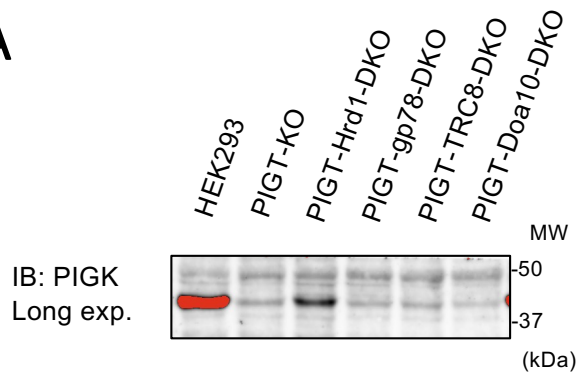**B**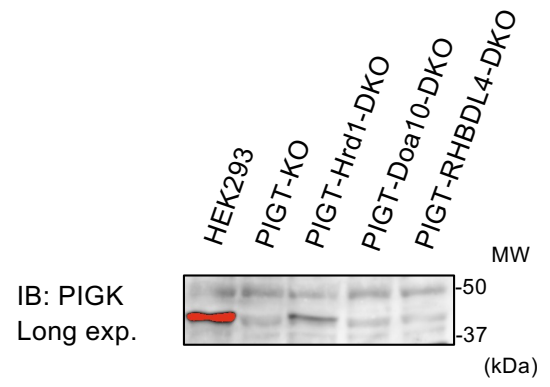**C**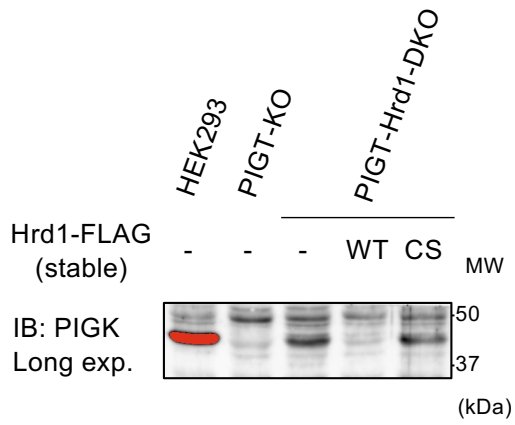**Fig. S1**

Supplement: Supplementary file 1 — Fig. S1 [file csf_46_21019_1.pdf]
